# Making theory explicit - An analysis of how medical education research(ers) describe how they connect to theory

**DOI:** 10.1186/s12909-016-0848-1

**Published:** 2017-01-19

**Authors:** Klara Bolander Laksov, Tim Dornan, Pim W. Teunissen

**Affiliations:** 1 Department of Learning, Informatics, Management and Ethics (LIME), Karolinska Institutet, Stockholm, Sweden; 2 Department of Education, Centre for the Advancement of University Teaching, Stockholm University, Stockholm, Sweden; 3 Dentistry and Biomedical Sciences, School of Medicine, Queen’s University Belfast, Belfast, UK; 4 School of Health Professions Education (SHE), Faculty of Health, Medicine and Life Sciences, Maastricht University, Maastricht, NL Netherlands; 5 Department of Obstetrics & Gynecology, Gynaecologist at VU University Medical Center, Amsterdam, The Netherlands

## Abstract

**Background:**

As medical education develops into a varied and well-developed field of research, the issue of quality research anchored in or generating theory has gained increasing importance. Medical education researchers have been criticized of not connecting their work to relevant theory. This paper set out to analyse how researchers can connect to theory in medical education. The goal of this paper is to provide an accessible framework for those entering medical education research, regarding how theory may become an integral part of one's work.

**Methods:**

Fifteen purposefully selected researchers in medical education were asked to nominate papers they considered influential in medical education. Through this process 41 papers were identified and included in the study.

**Results:**

The papers were analysed with thematic content analysis, which resulted in three approaches to the use of theory: as close-up exploration; as a specific perspective; and as an overview. The approaches are exemplified by quotes from the papers included in our dataset and further illuminated by a metaphoric story.

**Conclusions:**

We conclude by pointing at the importance of making explicit how theory is used in medical education as a way to collaboratively take responsibility for the quality of medical education research.

**Electronic supplementary material:**

The online version of this article (doi:10.1186/s12909-016-0848-1) contains supplementary material, which is available to authorized users.

## Background

For over a decade, there have been expressions of concern about medical education research publications lacking an explicit theoretical basis [1–5]. Although there are signs of an increase in use of theory in medical education [6], it is of interest to not only identifying the issue, but to better understand and remedy it. The aim of this paper is to help researchers make better use of theory by examining how people have done so in the past and suggesting how others might do so in the future. First, this requires an elaboration of what we mean by theory.

A general description of theory is that it is a system of ideas intended to explain a phenomenon. This perspective on theory is consistent with the view that is often taken in biomedical and physical research and is clearly linked to theory as something that can be repeatedly tested, and hence guide activity in all cases. However, theory in medical education needs to be viewed as different from the biomedical view. Rather than emphasising an imperative of proof [7], the point of departure is the participation in scientific dialogue around different explanations of phenomena with a specific lens through which the inquiry was conducted, which will result in theory generation [8]. Reeves and colleagues (ibid.) define theory as: *"an organized, coherent, and systematic articulation of a set of issues that are communicated as a meaningful whole”.* This definition is useful from a medical education as well as biomedical view on theory.

The conceptualisation of theory in education can be placed historically during the 20th century [9] at a continuum that covers different levels of abstraction ranging from high level theories at the turn of the 20th century, to middle range theories in the 1960s, and personal practice theories by the end of the 1900s. High level theories state the fundamental variables of systems and include a high level of abstractness, like Marxist theory, which are ’independent of the thing to be explained’ (social struggle, for example) to the extent that they might not arise from empirical research or lead directly, via testable ideas or hypotheses, to empirical research, however it can provide guidance for empirical enquiry.

In a seminal paper half a century ago, Merton (1968) introduced the idea that there are middle range theories *– theories that lie between the minor but necessary working hypotheses that evolve in abundance during day-to-day research and the all-inclusive systematic efforts to develop a unified theory that will explain all the observed uniformities of social behavior, social organization, and social change (*[10]*p. 39.).* Bordage argued that, in the medical education domain, programmatic research potentially leads to middle range theories [11]. This is an iterative process in which observations give rise to (or refine) a theory, which guides further empirical research, and which further refines the theory. At the most detailed and individual level, ’personal theories’ [12] guide the day-to-day activities of every one of us. Our choice of how to feedback on student performance, for example, is most often guided by a highly individual theory of how to communicate and appraise performance. It is a personal theory, which is in a two-way relationship with empirical observations, even if it only tells us when to say what and how in relation to the student. In education, Donald Schön’s (1991) research has focused on these so called theories-in-use, that teachers apply in everyday teaching, and how they relate to their ’espoused theory’, which could be mid-range theories of feedback and communication patterns together with course design that might have been learned in a faculty development course. The focus of this paper is on how middle-range theory can be made explicit, since the contribution to development of theory depends on how effectively the community of scholars ‘integrates inquiry frameworks to achieve practical relevance’ [13].

Whatever one’s paradigm, being clear about the theoretical assumptions that underly research adds value to it. When people call for medical education research to be better theorised, they are asking researchers to position their work within some explicit theoretical framework, be able to justify how and why they did so, and use insights derived from the framework to help interpret empirical observations. Moving from philosophical considerations to more practical ones, Bordage (2009) explained how education researchers can use conceptual frameworks as ‘ways of thinking about a problem or study, or a way of representing how complex things work.’ Such conceptual frameworks may guide researchers to look at problems in particular ways or generate hypotheses to be tested [14] and are thus crucial in the linkage between theory and empirical data. They may arise from their own or other people’s research and the conceptual framework can be derived from a specific theory. When theories are adopted by many different researchers, they help the field build up a coherent body of work, which is transferable beyond the conditions in which individual studies were conducted.

As teachers of education research methodology, we have consistently found that Masters students, PhD students, and new medical education researchers find theory a difficult topic to engage with. It seemed logical to help them, not by writing another abstract paper about theory, but by developing an understanding of how authors describe their theory use in medical education research publications. To achieve this, we decided to look at a sample of influential medical education publications, which could help us explain how connections to conceptual frameworks are formed and used, and provide exemplars that would help neophyte researchers bridge theory and practice explicitly.

## Methods

### Conceptual orientation

A social constructivist approach [15] guided our research [16–18]. Social constructivism assumes that groups or communities create shared meaning as a result of their interactions. These shared meanings can be attributed to things, which are called ’artefacts’, such as a journal or a position or title, and together contribute to a shared culture. In this project, the research was *social* in that we regarded publications as artefacts produced by the collaborative efforts of the medical education community. It was constructivist in that an iterative process of data analysis and theory development between the three authors allowed the construction of an interpretation of how connections to conceptual frameworks were formed and used in the publications we included as data.

### Data collection procedure

The dataset for this project consisted of a set of published papers that were deemed influential in the medical education domain. The data collection was carried out in two stages. The first stage included the use of a delphi-inspired approach where we, instead of selecting a set of papers based on our own interests or on potentially deceptive citation indices, based on our shared knowledge of the medical education field and its’ inhabitants, purposefully selected fifteen medical education researchers to nominate papers. In light of our aim, fifteen was considered a large enough number to allow for some dropout and still leave us with a large enough number, more than forty, of publications to analyse. The selected researchers differed in their variation of methodological preferences usually applied in their own research as well as research topic, gender and geography (see Table 1). They received the following request:

**Table 1:** Listing of variation among the scholars who nominated papers

Methodologies applied	phenomenological, grounded theory, discourse analysis, RCT studies, ethnography
Research topics	assessment, communication, clinical reasoning, professionalism, medical education research, team work, learning
Gender	8 men, 7 women
Geography	North America, Europe, Asia, Australia

- Please nominate approximately 5 research papers you consider as influential in the field of medical education. For each paper, please write a few sentences saying why you chose it.

The instruction to the scholars who nominated the papers were asked for papers that were considered influential to their work rather than papers that used theory in a particularly good way. By influential we clarified that it could be “*research papers that have, in your opinion, impacted medical education practice or research in general or your own research or educational practice*”. We deliberately chose the term ‘influential’, since this allowed the nominaters to choose papers whose research quality might be regarded as low, as well as high as not to require explicit criteria for quality, something we viewed problematic. The emphasis on influential rather than explicit theory use we believed also would provide us with a better data material in terms of the variation in how the use of theory was described. Unfortunately we did not receive enough responses as to why the researchers had chosen the research articles to be able to include that in our analysis.

### Analysis

The analysis of the papers was conducted iteratively based on principles of qualitative thematic content analysis [19]. The first step was a pilot exercise to develop a framework, which would be used for the main dataset. Three articles were selected to be read by all three researchers. Each wrote a short analysis of how theory was used in the three articles, which were discussed and led to the formulation of four main questions to guide the main analysis: 1) ”What was the starting point of this article?” The starting point could be, for instance, a practical or theoretical problem, or the findings of previous research. 2) ”What conceptual framework was used to approach the problem?”. This is where we could see a more or less explicit linkage to theoretical concepts or frameworks. 3)”How did the paper address the problem methodologically?”; Guba & Lincoln’s [20] typology of methodological approaches guided our analysis. 4)”How did the article contribute to theory?”. The questions were applied successfully to 6 more of the articles, following which we reviewed all interpretations in order to develop a set of heuristics that might be useful to other researchers. This resulted in a refined and elaborated set of questions, which we used to analyse the entire dataset:

1. What was the authors’ point of departure?
  - Where did the problem come from (e.g. practical issue, previous papers, theoretical problem, hypothesized based on theory)?
2. What route did the authors take?
  - How was the issue problematized and conceptualized?
  - How do the answers to questions 1 and 2 relate to each other?
  - What methodology did the authors use to tackle their problem and how explicit were they in considering their options?
  - Where did the authors arrive?
    - How did they suggest they had contributed to addressing the problem under investigation?
    - What is the apparent relationship between the different components of this scientific journey?

Although use of theory was not always described explicitly, from our social constructivist perspective, theory was implicitly or explicitly described, sometimes only as assumptions, and could be seen as a way of talking, a discourse, that was employed in the article. These assumptions were, from our perspective, stemming from the shared understanding in the medical education community of how certain methods or analysis procedures are understood to be of value.

This analytic framework was then applied to the entire dataset. All articles were read by two of us and we entered our answers into a web based database. Discussion of similarities and differences identified three linked metaphors of how theory was used in the included articles. Finally, we read all papers again and mapped them to the metaphors.

## Results

Ten of the 15 invited researchers, six men and four women, nominated a total of 41 papers. Two declined the invitation and three did not reply. The papers are listed in Additional file 1: Appendix 1 and ranged from empirical papers, to reviews, conceptual papers and editorials. As the nominating researchers were asked to suggest influential research papers, we did not want to omit papers based on our own judgement of what counts as a research paper and hence decided to include all the nominated papers regardless of type of publication. We identified three distinct, but sometimes overlapping, ways in which authors connected to existing theory: a close-up exploration; a specific perspective; and a distanced perspective. We explain each theme below, followed by examples to make the meaning of each category more substantial.

## Close-up exploration

Here, researchers aimed to explain some specific phenomenon, such as how residents learn from practical experience. Either instigated by a local issue or issues raised in other studies, they recognized a need or opportunity to add to the current understanding of this phenomenon. This allowed them to formulate a specific question, decide on a research plan, and set out to do the research. Middle range theory contributed to this process by helping them choose questions, methods, and a setting in which to conduct the research, which would contribute to building a clearer or novel understanding of the topic of interest.

### Example 1

An example of a study in this category is a study by Lingard et al. (2004). Examining communication failures in operating rooms, Lingard and colleagues [21] took as their point of departure an issue stemming from previous research:

Recent evidence suggests that adverse events resulting from error happen at unacceptably high rates in the inpatient setting and that ineffective or insufficient communication among team members is often a contributing factor. (p.330)

By referring to a growing body of literature regarding the relationship between teamwork and safety in health care, and trends in the way it had been studied, the route taken by Lingard et al. identified a gap of knowledge:

While these models have reinforced the importance of communication in effective team function, their multidimensionality precludes in depth attention to the individual variable of communication. (p.330)

The authors continued by referring to the findings from studies on communication in the specific context of operating rooms, formulated as ”lack of standardization and team integration”. Here, they referred to the ways of thinking about communication in aviation industry (i.e. theoretization from another field) both as a way to frame the issue at hand (communication failures) and to choose interventions to overcome the problem:

One potential solution to the described weaknesses in OR team communication is to adapt the checklist system currently in use for systematic preflight team communications in the aviation industry … we anticipate that a carefully adapted checklist system could promote safer, more effective communications in the OR team. (p.330)

The methods section aligned with the methodological gap identified at the outset of the paper and used a theory-based framework for analysis of the fieldnotes taken of the communication that was observed. This enabled the researchers to approach and identify the characteristics of communication failures and arrive at a more detailed understanding of the topic under exploration. It allowed them to analyse these failures in relation to the effects at system, process, and patient level and arrive at a detailed understanding of the landscape under investigation: communication in the operating room.

### Example 2

Another example of the first category is the study by Van Zanten [22]. It starts with an overview of existing knowledge on the topic of patient satisfaction in relation to physician ethnicity. The authors summarize what other people have discovered, not to reconceptualise the scientific landscape but to explain what part of it they want to explore and what they expect to find:

“While there are a number of identifiable standardized patient (SP) and physician characteristics that may influence the satisfaction ratings provided to the candidates, the purpose of this study was to look at possible differences in satisfaction ratings as a function of candidate and SP ethnicity. It was hypothesized that, given the simulated environment, the interaction of candidate and SP ethnicity would not significantly impact the ratings given to candidates. Evidence to the contrary, while in accord with most actual doctor–patient-encounter research studies, would suggest that the satisfaction ratings may be influenced by factors not related to the abilities of the candidates.” (p.15)

Van Zanten et al. (2004) asked trained standardized patients in a performance-based examination of international candidates applying for graduate entry to the United States to rate their overall satisfaction with physicians on five-point scales. The article shows that SPs generally gave higher satisfaction ratings to encounters in which there was racial concordance which the authors linked to the findings of other researchers.

## A specific perspective

This category included research that intended to add to theory buiding from a deliberately chosen, fixed vantage point. Researchers argued for the advantages of applying a particular research perspective derived from psychological, sociological, anthropological, or philosophical domains to an issue in the field of healthcare education.

### Example 1

Albert and colleagues [2] interviewed a number of key figures from the English-speaking medical education community to inform the ongoing debate on what types of research should be accepted for publication in medical education. They introduced Bourdieu’s concept of ’field’ as a specific perspective from which to shine light on medical education research. Building on the cartographic metaphor introduced in the previous section, they used their Bourdieuvian perspectives to uncover particular aspects of the rocky landscape of medical education, as represented in empirical data. As a result, they were able to define ways of improving the quality of research in medical education. In that way, Albert et al. used the Bourdieuvian lens of ’field’ to bring weaknesses in medical education research to the surface.

### Example 2

The practical problem addresssed by Kerosuo and Engeström [23] was provision of care by multi-professional groups. They set out to examine how people in organisations learned to work collectively. They took a Change Laboratory approach, informed by Activity Theory, a theory that seeks to understand human activities as systemic and socially situated phenomena and hence bridges the gap between the individual subject and the social reality, to understand and change the health care environments they were working in.

Change Laboratory is an interventionist methodology for work-based learning and development of the activity. In the currently analyzed data, ten medical doctors and nurses in the pilot group applied the new calendar, care map and care agreement tools in the care of one of their patients. These tools are conceptualized as potential integrated instrumentality for the patient care.

The researchers arrived at a description of common themes in the implementation process. They expanded notions about organizational learning by asserting that it also involved tool-creation and implementation of these tools. Here, the methodological approach of the change laboratory became the lens through which the project was designed, carried through and interpreted.

## A distanced perspective

This third category operates at a relatively abstract level. Scholars scan an area of research, piecing together what others had previously mapped and identifying contradictions and areas that need further exploration. It would not be possible to do this type of work were it not for the efforts of researchers who have done close up explorations of specific phenomena or looked at the issue from a specific perspective. However, sometimes one needs to take a step back and look at how the pieces of information fit together, or not. Typically, papers in this third category do not report new empirical data; instead, previous research findings are their data.

### Example 1

A systematic review by Steinert et al. [24] on faculty development started from the observation that a myriad of faculty development programs had been delivered without any clear understanding of differences in their effectiveness. By scanning the numerous pieces of knowledge produced by other scholars, the authors were able to map out how these pieces fitted together, overlapped, and left areas undiscovered. This led to a conceptual framework that synthesized the knowledge generated by previous research.

This framework acknowledges the different roles of faculty members, of which teaching is one. It also highlights the fact that many mediating factors beyond specific faculty development activities can influence teacher effectiveness, and that outcome can be observed at a number of levels. (p.500)

The authors used evidence about faculty development to produce a framework that contributes to people’s thinking about their actions.

### Example 2

This example is provided by Schmidt, Norman & Boshuizen [25], who concluded from a review of literature on clinical competence:

…a number of recurrent problems emerged, casting doubt on some of the fundamental assumptions about the nature of clinical competence. (p.611)

By taking an overview, they were able to produce a novel conceptualization of clinical skills, which explained existing research findings.

The theory we elaborate here rests on three assumptions. First, in acquiring expertise in medicine, students progress through several transitory stages, characterized by distinctively different knowledge structures underlying their performances. Second, these representations do not decay or become inert in the course of developing expertise but rather remain available for future use when the situation requires their activation. Third, experienced physicians, while diagnosing routine cases, are operating upon knowledge structures that we call ”illness scripts”. (p.613)

An organizational framework – presented by the authors as a theory - was thus laid out and illustrated with cases, which showed how problem solving develops. The example above is based on a literature review. In other words, when theory is approached as overview, some form of literature review is required, but there is a difference in whether the aim is to provide guidance in terms of how practice should change and/or to provide and develop theory.

## Discussion

Having analysed 41 medical education research articles, we propose that there are three qualitatively different ways of connecting with theory, defined as a system of ideas intended to explain a phenomenon. However, although the three approaches were discerned from our primary data (the papers), there were papers that could not easily be categorized into only one of the categories. This was mostly due to the fact that these papers had not made their theoretical point of departure explicit. There were also examples of publications in the form of commentaries that did not fit any of the approaches.

As well as a categorisation, our analysis has produced a metaphor, which we hope will help explain how theory is used. The metaphor is of a person wanting to explore a coastal landscape and being able to do so from a boat, a lighthouse, or a plane. The coastal landscape represents the people, their behaviour, and the social processes that together constitute a field of inquiry. The boat, lighthouse, and plane provide three different perspectives, levels of detail, and types of illumination of the landscape. This ’story’ is outlined below.

### A narrative explaining the system of metaphors used in this paper

*Imagine you have to chart a far-off island. There are some crude, inaccurate maps of it made by people who lived there in the distant past. At a vantage point on the island stand the solitary remains of a lighthouse. The island is being surveyed because there may be valuable mineral deposits there. You have, at your disposal, three ways of surveying it. You can approach its rocky coast by boat, you can survey it from the top of the lighthouse or you can overfly it.*

*According to this metaphor, the island is a research topic. The valuable mineral deposits are a purpose for surveying it. The map represents the state of knowledge of the topic. The boat, lighthouse, and plane represent the three different ways theory can help refine the map discussed in the finding section: theory as close-up exploration (boat); theory as a specific perspective (lighthouse); and theory as overview (plane). You would get very different types and levels of detail, and perspectives on the rocky landscape from them. In the same way, the crude map you inherited was influenced by the perspective from which the land was surveyed and the sophisticated map you produce will, likewise, be influenced by the perspective you have chosen as well as the topographical features of the island.*

*This metaphor illustrates a fundamental principle about research. There is no single, incontrovertible way of knowing a topic, just as there is no incontrovertible way of knowing a landscape. Whether we acknowledge it or not, “truths”, like maps, are influenced by the theoretical perspective from which they were gleaned. Ultimately, theory permeates our research in many ways, just as perspective and distance leaves their indelible marks on a map. Even the decision that a topic is, like mineral deposits, worth exploring is influenced by theory. But let’s stick with those three different perspectives and how they can help you achieve your goal.*

*The boat allows you to come close to the landscape; even touch it. You can get very fine detail. It would be invaluable if, for example, you wanted to plan where to build a dock for ships exporting the valuable mineral. But it would not be so good for putting the entire island into a coherent perspective. In research terms, this use of theory means identifying a specific area of interest, getting out there and investigating a specific piece of the map. It is better at giving fine detail of part of a topic than producing a coherent map of the topic as a whole. Surveying solely by boat could produce a patchy map of the field of interest with unresolved, conflicting results.*

*You would choose the lighthouse if its fixed vantage point helped you, for example, choose the route from the mineral mine to the dock across an undulating landscape. Likewise, theory can help you add a piece of information to the evolving map of scientific knowledge from the deliberately chosen, fixed vantage point. You might choose some specific psychological, sociological, anthropological or philosophical stance because you want to know what that stance will tell you about the topic. Having done so from the lighthouse, you would shed a valuable new perspective on a topic, though perhaps not at the same level of detail as if you had surveyed it from a boat.*

*A plane allows you to overview the entire landscape and, for example, pull together the previous efforts of surveyors in boats and lighthouses into a more or less fitting whole. As a researcher, the plane perspective could help you identify misrepresentations or areas that need further exploration, though it would not allow you to examine topics in the same detail as either a lighthouse or a boat. It could give you valuable insights into the state of the maps so far and provide new insights that help drive future research agendas.*

By applying the metaphors, strengths and weaknesses attached to different ways of using theory in medical education research can be uncovered. Being in a boat limits, for example, the scope of the quest; it works best when focusing on one question at a time in a well-defined area. The result of many researchers trying to answer different but related questions is a patchy map of the field of interest, with areas that are well defined, blind spots, and conflicting findings. The lighthouse perspective can be used to look at different areas and study their peculiarities. Areas that were previously researched by boat can be re-examined and this can lead to valuable enlightenment. However, researching the world from a lighthouse comes at the expense of flexibility. Areas on which the chosen perspective does not shed light cannot be explored because the lighthouse cannot move around a research topic like a boat can. It is thus essential that lighthouse researchers describe the perspective thoroughly and acknowledge that using a different perspective (or light) might have been brought forward different findings. The plane approach provides an important resource to the research community by generalising and building on or critiquing different people’s work. However, the distance from the area of interest results in loss of detail. On the other hand, the oversight one gets from being in a plane can lead to valuable outlines of the state of the map so far and even result in new insights that drive future research agendas.

## What this study adds

So, how do the three perspectives of how theory is made explicit inform our understanding of theory? Theory is not an automated result of empirical research but emerges from a choice on the part of the researcher [26]. In contrast to theory use in the positivist paradigm applied in biomedical research, where the function of theory is as a tool in generating research ideas and predict outcomes in empirical studies we have in this study exemplified the use of theory in medical education by the three approaches. Together we suggest that they provide a basis for understanding the development and use of theory at the mid-range theory level allthough we acknowledge the boundaries between personal theory, mid-range and high level theory to be blurred.

By analysing how theory was approached in the articles we could see a variation in approaches. Firstly, we could see that the included articles approached theory ranging from micro-level theory to mid-range theory [10]. Secondly we saw a difference in the degree to which the articles worked with theory to better understand a phenomenon, i.e. generated research questions, methodology and interpretation at one end, or contributed to theory as a result of an inductive process of data analysis, at the other. As several of the papers were based on a practical problem, the paper specifically aimed to answer this specific question, and did not intentionally also contribute to mid-range theory. Here, often, theory was only viewed as findings from previous research. However, there were also examples where the research question was framed in relation to theory, where the research question was based on particular theories, and the paper is an example of an argumentation in relation to that theory, and as such is a contribution to a theoretical discussion. Finally there was a difference in the way in which theory was introduced in a paper. This ranged from very subtle or implicit introduction of theoretical stance, to very clear and conceptual explanations of the theoretical perspective. If we go back to the definition of theory referred to in the background [8] it is less helpful when theory is not made explicit. Although it was possible to read between the lines in terms of the theoretical stance taken by some authors of the publications, it became clear that papers where theory was made explicit were participating in a scientific dialogue with a specific lens, rather than claiming to having found proof of something, in a technical sense. As theory is gaining in importance [27], and should continue doing so, the way of introducing and discussing theory as part of the research process is vital.

Already in 1968, Habermas emphasized the need for diversity in how theory, or knowledge interests as he labels the different approaches to knowledge and theory, is approached [28]. Different approaches are necessary and in play to different degree in different disciplines or scientific tradtions. Adapted to medical education, it seems that although to a large degree being a social science, the aim for establishing objective truths has for a long time dominated the research, something that is also part of our findings as exemplified by studies written in a (post) positivist tradition. However, several of the papers included in this study challenged this view and papers including both hermeneutic and emancipatory knowledge interests were also included.

In a contribution by Hodges and Kuper [29], three different kinds of theory are outlined as useful for graduate medical education; bioscience theories, learning theories and socio-cultural theories. The authors emphasize the merits of anchoring medical education problems to theory and elaborate on what the different theoretical stances have to offer as well as their weaknesses. Consequently we believe the current article adds insight into how theories are used. The paper itself is a worked example of how researchers can learn from specifically looking at papers from this standpoint.

A number of guiding articles to researchers who are new to the field of medical education research already exist. One such article is ’The research compass’ [30], in which readers are guided through four categories of research approach; explorative, experimental, observational and translational studies. A main point made in that paper is that research should be about researchable problems that lead to generalisable knowledge and are practically relevant. By asking simple questions and use simple methods, the approaches *theory as close-up exploration* and *theory as specific perspective* play a crucial role both in terms of providing a scholarly approach to the development of teaching and learning (scholarship of teaching and learning), and in providing the basis for the development of theory at higher level as when theory is used *as overview*. Finally, Thomas [26] argues for the need for more ’bricolage’ in educational enquiry, giving room for multiple theoretical approaches in exploring the field of research. This need for multiple perspectives was recently commented as increasing in medical education [27] and it is in line with our view that research with *theory as a specific perspective* is increasing in medical education.

## Methodological considerations

Our description of how theory is used in medical education has limitations. The starting point for our analysis was articles nominated by highly regarded researchers representing different paradigmatic stances as a point of departure. Had we, instead chosen to ask a random group of, less experienced researchers, or a different set of papers, for instance based on citation indices or just picked a random sample, the outcomes of this study may have been different. We therefore acknowledge the nomination process as an important aspect of the data generated as it provided a broader scope in terms of the publications included as data in the study.

Secondly, uncovering the way in which researchers use theory in their work based on an anlysis of papers may paint a distorted image. Papers are written to convey the research findings and their implications, not the way in which researchers interacted with theory. Our use of theory may not meet the intentions of the authors of the papers that were discussed in the study.

Thirdly, as this study aimed to explore variation in how connections to conceptual frameworks are formed and used, we treated each article as a separate representation. It was hence not included in our scope to explore whether any of these approaches was more common than the others.

## Conclusion

The continuous criticism of medical education research as a field that lacks theoretical basis is subject to decreased justification. As it is an area characterized by research carried out by researchers from multiple disciplinary and paradigmatic backgrounds the assumptions of how to treat the issue of theory in medical education research will probably be contentious depending on the perspective one brings to research. At a minumum, we argue, theory use needs to be made explicit. The current paper shows three different approaches to how to connect to theory in medical education research; as close-up exploration, as specific perspective and as overview. Suggestions are given as to how researchers new to the field of medical education can clarify their theoretical basis.

## Additional file

Additional file 1: List of publications. Here the list of publications that was the result of the nomination process are listed. (DOC 46 kb)
