# Supplementary material for: Making theory explicit - An analysis of how medical education research(ers) describe how they connect to theory
Source: BMC Med Educ. 2017 Jan 19;17:18. doi: 10.1186/s12909-016-0848-1 (PMC5248446; doi:10.1186/s12909-016-0848-1)
Supplement: Additional file 1: — List of publications. Here the list of publications that was the result of the nomination process are listed. (DOC 46 kb) [file 12909_2016_848_MOESM1_ESM.doc]

Appendix: list of publications

Albanese, M.A., and Mitchell, S. (1993) Problem-based Learning: A Review of Literature on Its Outcomes and Implementation Issues. *Academic Medicine* 68(1) 52-81

Albert, M., Hodges, B., & Regehr, G. (2007). Research in medical education: Balancing service and science. *Adv Health Sci Educ Theory Pract*, *12*, 103–115.

Brosnan, C. (2010). Making sense of differences between medical schools through Bourdieu’s concept of “field”. *Medical education*, *44*(7), 645–52. doi:10.1111/j.1365-2923.2010.03680.x

Colliver, J. a. (2000). Effectiveness of problem-based learning curricula: research and theory. *Academic medicine : journal of the Association of American Medical Colleges*, *75*(3), 259–66. Retrieved from http://www.ncbi.nlm.nih.gov/pubmed/10724315

Cook, D. a, Bordage, G., & Schmidt, H. G. (2008). Description, justification and clarification: a framework for classifying the purposes of research in medical education. *Medical education*, *42*(2), 128–33. doi:10.1111/j.1365-2923.2007.02974.x

Davenport, N. H. M. (2011). Medical residents’ use of narrative templates in storytelling and diagnosis. *Social science & medicine (1982)*, *73*(6), 873–81. doi:10.1016/j.socscimed.2011.01.036

Friedman, V. J. (2001). Designed Blindness: An Action Science Perspective on Program Theory Evaluation. *American Journal of Evaluation*, *22*(2), 161–181. doi:10.1177/109821400102200203

Ginsburg, S., Regehr, G., Hatala, R., McNaughton, N., Frohna, A., HODGES, B., LINGARD, L., et al. (2000). Context, conflict, and resolution: a new conceptual framework for evaluating professionalism. *Academic Medicine*, *75*(10), S6. Retrieved from http://www.ncbi.nlm.nih.gov/pubmed/11031159

Gleeson, F. A. (n.d.). Medical Education Booklet No. 8 Assessment of clinicbl competence using an objective structured clinical examination (OSCE). *Medical Education*, (8).

Gruppen, L. D. (2007). Is medical education research “hard” or “soft” research? *Advances in Health Sciences Education*, *13*(1), 1–2. doi:10.1007/s10459-007-9092-0

|  |
| --- |
|  |

Hafferty, F. W., & Franks, R. (1994). The hidden curriculum, ethics teaching, and the structure of medical education. *Academic Medicine*, *69*(11), 861-71.

Harden, R. M., Crosby, J. R., Davis, M. H., & Friedman, M. (1999). AMEE Guide No . 14 : Outcome-based education : Part 5Ð From competency to meta-competency : a model for the speci ® cation of learning outcomes, *21*(6).

Hauer, K. E., Ciccone, A., Henzel, T. R., Katsufrakis, P., Miller, S. H., Norcross, W. a, Papadakis, M. a, et al. (2009). Remediation of the deficiencies of physicians across the continuum from medical school to practice: a thematic review of the literature. *Academic medicine : journal of the Association of American Medical Colleges*, *84*(12), 1822–32. doi:10.1097/ACM.0b013e3181bf3170

Heaven, C., Clegg, J., & Maguire, P. (2006). Transfer of communication skills training from workshop to workplace: the impact of clinical supervision. *Patient education and counseling*, *60*(3), 313–25. doi:10.1016/j.pec.2005.08.008

Hodges, B. (2006). Medical education and the maintenance of incompetence. *Med Teach*, *28*, 690–696.

Hodges, B. (2005). The many and conflicting histories of medical education in Canada and the USA: an introduction to the paradigm wars. *Medical education*, *39*(6), 613–21. doi:10.1111/j.1365-2929.2005.02177.x

Hodges, B., Regehr, G., McNaughton, N., Tiberius, R., & Hanson, M. (1999). OSCE checklists do not capture increasing levels of expertise. *Academic Medicine*, *74*(10), 1129-34.

Kerosuo, H., & Engeström, Y. (2003). Boundary crossing and learning in creation of new work practice. *Journal of Workplace Learning*, *15*(7/8), 345–351. doi:10.1108/13665620310504837

Kneebone, R., Nestel, D., Yadollahi, F., Brown, R., Nolan, C., Durack, J., Brenton, H., et al. (2006). Assessing procedural skills in context: Exploring the feasibility of an Integrated Procedural Performance Instrument (IPPI). *Medical education*, *40*(11), 1105–14. doi:10.1111/j.1365-2929.2006.02612.x

Lingard, L. (2004). Communication failures in the operating room: an observational classification of recurrent types and effects. *Quality and Safety in Health Care*, *13*(5), 330–334. doi:10.1136/qshc.2003.008425

Lingard, L., Reznick, R., DeVito, I., & Espin, S. (2002). Forming professional identities on the health care team: discursive constructions of the “other” in the operating room. *Med Educ*, *36*, 728–734.

Lingard, L., Reznick, R., Espin, S., Regehr, G., & DeVito, I. (2002). Team communications in the operating room: talk patterns, sites of tension, and implications for novices. *Academic medicine : journal of the Association of American Medical Colleges*, *77*(3), 232–7. Retrieved from http://www.ncbi.nlm.nih.gov/pubmed/11891163

Mamede, S., Schmidt, H. G., & Penaforte, J. C. (2008). Effects of reflective practice on the accuracy of medical diagnoses. *Medical education*, *42*(5), 468–75. doi:10.1111/j.1365-2923.2008.03030.x

Martimianakis, M. A., Maniate, J. M., & Hodges, B. D. (2009). Sociological interpretations of professionalism. *Medical education*, *43*(9), 829–37. doi:10.1111/j.1365-2923.2009.03408.x

Mcmanus, A. I. C., Smithers, E., Partridge, P., Keeling, A., Fleming, P. R., & Mcmanus, I. C. (2012). BMJ Publishing Group A Levels And Intelligence As Predictors Of Medical Careers In Uk Doctors : 20 Year Prospective Study Learning in practice A levels and intelligence as predictors of medical in UK doctors : 20 year prospective study. *Group*.

Moulton, C. E., Regehr, G., Mylopoulos, M., & MacRae, H. M. (2007). Slowing down when you should: a new model of expert judgment. *Academic medicine : journal of the Association of American Medical Colleges*, *82*(10 Suppl), S109–16. doi:10.1097/ACM.0b013e3181405a76

Moulton, C., Regehr, G., Lingard, L., Merritt, C., & MacRae, H. (2010). Slowing down to stay out of trouble in the operating room: remaining attentive in automaticity. *Academic medicine : journal of the Association of American Medical Colleges*, *85*(10), 1571–7. doi:10.1097/ACM.0b013e3181f073dd

Norman, G. R. (1999). The adult learner: a mythical species. *Acad Med*, *74*, 886–889.

Norman, G. (2003). RCT = results confounded and trivial: the perils of grand educational experiments. *Medical education*, *37*(7), 582–4. Retrieved from <http://www.ncbi.nlm.nih.gov/pubmed/12834412>

Norman, G. R., & Schmidt, H. G. (1992). The psychological basis of problem-based learning: a review of the evidence. *Academic medicine*, *67*(9), 557-65.

Pawson, R., Greenhalgh, T., Harvey, G., & Walshe, K. (2004). Realist synthesis: an introduction. *ESRC Research Methods Programme. Manchester: University of Manchester*. Retrieved from http://www.ccsr.ac.uk/methods/publications/RMPmethods2.pdf

Robb, N., Dunkley, L., Boynton, P., & Greenhalgh, T. (2007). Looking for a better future: identity construction in socio-economically deprived 16-year olds considering a career in medicine. *Social science & medicine (1982)*, *65*(4), 738–54. doi:10.1016/j.socscimed.2007.03.011

Savoldelli, G.L., Naik, V.N., Park, J., Joo, H.S., Chow, R. & Hamstra, S.J. (2006). Value of Debriefing during Simulated Crisis Management: oral versus video-assisted oral feedback *Anesthesiology*, 105(2), 279–285.

Schmidt, H., Norman, G., & Boshuizen, H. (1990). A cognitive perspective on medical expertise: theory and implication [published erratum appears in Acad Med 1992 Apr; 67 (4): 287]. *Academic medicine, 65*(10), 611-621.

Steinert, Y., Mann, K., Centeno, A., Dolmans, D., Spencer, J., Gelula, M., & Prideaux, D. (2006). A systematic review of faculty development initiatives designed to improve teaching effectiveness in medical education: BEME Guide No 8. *Med Teach*, *28*, 497–526.

Stern, D. T. (1998). Practicing what we preach? An analysis of the curriculum of values in medical education. *The American journal of medicine*, *104*(6), 569–75. Retrieved from http://www.ncbi.nlm.nih.gov/pubmed/9674721

Taylor, J. S. (2003). Confronting “‘Culture’” in Medicine’s “‘Culture of No Culture’.” *Western Medicine*, 555–559.

van der Vleuten, C. P. M., & Schuwirth, L. W. T. (2005). Assessing professional competence: from methods to programmes. *Medical education*, *39*(3), 309–17. doi:10.1111/j.1365-2929.2005.02094.x

van Zanten, M., Boulet, J. R., & McKinley, D. W. (2004). The influence of ethnicity on patient satisfaction in a standardized patient assessment. *Academic medicine : journal of the Association of American Medical Colleges*, *79*(10 Suppl), S15–7. Retrieved from <http://www.ncbi.nlm.nih.gov/pubmed/15383378>

Vernon, D.T.A. & R.L. Blake (1993) Does problem based learning work? A meta analysis of evaluative work *Academic Medicine* 68 (7) 550-563

Woolf, K., Cave, J., Greenhalgh, T., & Dacre, J. (2008). Ethnic stereotypes and the underachievement of UK medical students from ethnic minorities: qualitative study. *BMJ*, *337*.
